# Challenges in long-oval canal cleaning: effects of instrumentation kinematics and ultrasonic irrigation

**DOI:** 10.1590/0103-644020256582

**Published:** 2026-01-19

**Authors:** Sandra Soares Kühne Busquim, Laila Gonzales Freire, Iandara de Lima Scardini, Marcelo dos Santos

**Affiliations:** 1 Private Practice, Vienna, Austria; 2 Department of Restorative Dentistry, School of Dentistry, University of São Paulo, São Paulo, Brazil

**Keywords:** accumulated hard-tissue debris, microcomputed tomography, root canal, smear layer, ultrasonic irrigation

## Abstract

The present study aimed to evaluate, using micro-CT imaging, the preparation of long-oval root canals performed by Twisted File Adaptive (TFA) and Reciproc (RC) instruments and the cleaning efficacy of final ultrasonic irrigation (UI) using a correlative scanning electron microscopic (SEM) approach. Distal long-oval canals of thirty-eight lower molars were divided into two groups (n=19): TFA and RC groups. Each specimen underwent three scans: pre- and post-instrumentation and post-UI, and changes in volume, non-prepared surfaces, and accumulated hard-tissue debris (AHTD) were measured. Then, root canals were halved at the apical third and analyzed under SEM for smear layer (SL) evaluation. Mann-Whitney and Kruskal-Wallis tests were used for statistical analysis (p<0.05), and the correlation between the AHTD and the SL was evaluated with Pearson's correlation coefficient. The TFA system exhibited greater efficiency in dentin removal compared to the RC system when considering the entire canal (p<0.05), with approximately 26 to 29% of surface area unprepared for the whole canal (p>0.05). Both systems produced the same volume of AHTD (p>0.05). Final UI led to a notable reduction in AHTD (60-70%), except in the apical third. Qualitatively, SEM analysis revealed non-uniform dentin remnants covering the canal walls, particularly prominent in the RC group. The data analysis determined a strong correlation between AHTD and SEM evaluation. In conclusion, neither system achieved complete preparation of long-oval root canal walls, and the final UI reduced AHTD. The AHTD observed by micro-CT was related to the presence of SL evaluated by SEM.

## Resumo

O presente estudo teve como objetivo avaliar, através de imagens de micro-CT, o preparo de canais radiculares ovais-longos utilizando os instrumentos Twisted File Adaptive (TFA) e Reciproc (RC) e a eficácia da limpeza da irrigação ultrassônica (IU) através de uma abordagem de microscopia eletrônica de varredura (MEV) correlacional. Canais distais ovais-longos de trinta e oito molares inferiores foram divididos em dois grupos (n = 19): grupos TFA e RC. Cada espécime foi submetido a três varreduras: pré e pós-instrumentação e pós-IU, e mudanças no volume, superfícies não preparadas e detritos de tecido duro acumulados (DTDA) foram medidos. Os canais radiculares foram divididos ao meio no terço apical e analisados em MEV para avaliação da camada de esfregaço (CE). Os testes de Mann-Whitney e Kruskal-Wallis foram utilizados para análise estatística (p < 0,05), e a correlação entre a DTDA e o CE foi avaliada com o coeficiente de correlação de Pearson. O sistema TFA exibiu maior eficiência na remoção de dentina em comparação ao sistema RC ao considerar todo o canal radicular (p < 0,05), com aproximadamente 26 a 29% da área de superfície não preparada para todo o canal (p> 0,05). Ambos os sistemas produziram o mesmo volume de DTDA (p> 0,05). A IU final levou a uma redução notável nos DTDA (60-70%), exceto no terço apical. Qualitativamente, a análise por MEV revelou remanescentes de dentina não uniformes cobrindo as paredes do canal, particularmente proeminentes no grupo RC. A análise dos dados determinou uma relação positiva moderada entre DTDA e avaliação por MEV. Concluindo, nenhum dos sistemas atingiu o preparo completo das paredes ovais longas do canal radicular, e a IU final reduziu os DTDA. Os DTDA observados por micro-CT foram relacionados à presença da CE avaliada por MEV.

**Figure:**
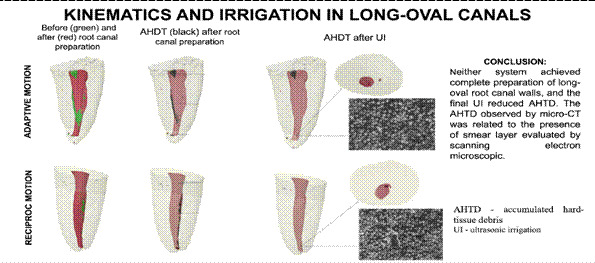

## Introduction

The anatomy of long-oval canals represents a challenge for adequately preparing the root canal walls during endodontic treatment ^1^ ^,^ ^23^. The potentially still infected unprepared areas provide a substrate for bacterial growth, compromising the endodontic treatment success ^4^ ^,^ ^5^. Furthermore, during root canal treatment, the action of the endodontic instrument may generate debris that can accumulate in anatomical eccentricities, especially in polar areas ^6^ ^,^ ^7^. To eliminate this debris formed by the remaining pulp tissue, bacteria and their by-products, and dentinal debris, which comprises organic and inorganic tissue, it is necessary to enhance the irrigation during root canal treatment ^4^ ^,^ ^6^.

In the last decade, important modifications in the design and kinematics of endodontic instruments have been proposed to improve cutting and cleaning performances and decrease the percentage of unprepared canal surfaces ^8^. The Twisted File Adaptive system (TFA) (Kerr, Brea, CA, USA) combines continuous rotation and reciprocating motion in a hybrid kinematic, adjusted automatically by a specific motor according to the load applied to the instrument. According to the manufacturer, the Twisted File technology means the file is twisted into shape for improved file durability, features R-Phase technology to improve file flexibility, and provides better debris removal ^9^ ^,^ ^10^ ^,^ ^11^. The reciprocating system (RC) (VDW, Munich, Germany) works in a reciprocating kinematic, removing a significant amount of dentin from the canal wall. However, due to a wider cutting angle and a smaller releasing angle, its flutes tend to push the dentinal debris towards the apex ^12^ ^,^ ^13^.

Micro-computed tomography (micro-CT) imaging is considered the most reliable method for analyzing morphometric changes in the root canal and quantifying the accumulated hard-tissue debris (AHTD) after endodontic treatment ^14^ ^,^ ^15^ ^,^ ^16^. Nevertheless, micro-CT assessment only covers hard tissues. Therefore, it is suggested that complementary methodologies be used to investigate the presence of the smear layer on the root canal wall after root canal instrumentation ^17^ ^,^ ^18^. To the best of the authors' knowledge, no literature has explored the relationship between AHTD volume observed via micro-CT and the presence of a smear layer examined through Scanning Electron Microscopy (SEM). Furthermore, more studies are needed to compare the efficacy of instrumentation in long-oval canals using TFA and RC systems.

This study aimed to evaluate: (i) the preparation of long-oval distal canals of mandibular molars and quantify the AHTD comparing TFA and RC systems, using micro-CT imaging; (ii) the effect of final ultrasonic irrigation (UI) in the removal of these AHTD; and (iii) the correlation between the presence of AHTD observed via micro-CT and the smear layer evaluated through SEM. The null hypothesis was that the different endodontic systems and the UI did not affect root canal preparation and the removal of AHTD, respectively.

## Materials and methods

### Teeth Selection and Sample Preparation

The sample size was calculated using G*Power 3.1 software for Windows (Heinrich-Heine-Universität ¨t, Düsseldorf, Germany). The ANOVA fixed effects: omnibus, one-way test was selected from the F-tests family to achieve a testing power beta of 80% with α = 0.05. Thirty-eight specimens (nineteen teeth per test group) were determined as the ideal sample size to observe significant differences. Following the approval of the local Ethics Committee (CAEE: 56448616.0.0000.0075), a sample of eighty extracted permanent mandibular molars was provided by a Biobank of Human Teeth. The teeth were scanned to assess root canal anatomy at an isotropic resolution of 17.42 µm using a micro-CT scanner (SkyScan 1176; Bruker microCT, Kontich, Belgium) at 90 kV, 278 µA, 360° rotation, and a 0.5° rotation step. The images were reconstructed into cross-sectional slices using NRecon v. 1.7.1.6 software and analyzed with CTan v.1.12.0.0 software (Bruker microCT, Kontich, Belgium). From this sample, 38 teeth were chosen based on specific inclusion criteria: distal roots presenting no previous endodontic treatment, a single patent large root canal - Vertucci’s type I ^14^ (confirmed by the passive insertion of a #K30 instrument), curvature less than 20°, and long-oval configuration ^19^ evaluated at 5 mm from the apex. The mesial root of each tooth was removed with a diamond disc and then returned to the Biobank of Human Teeth for potential use in future studies.

### Root canal preparation

The root length was standardized in 14 millimeters using a diamond disc. The root canal was explored with a #25 K-File until the tip was visible at the apical foramen. From this measurement, 0.5mm was subtracted to establish the working length (WL). Samples that #25 K-File did not reach the WL were substituted. For cleaning and shaping, the roots were covered with two layers of nail varnish and then embedded in polyvinyl siloxane (Perfil, Vigodent, Rio de Janeiro, Brazil) to reproduce the periodontal ligament and prevent passive irrigant extrusion ^20^. The specimens were divided into two groups according to the system used for the root canal preparation: TFA and RC (n=19). A single trained operator (S.K.B) with 15 years of experience performed all the preparation procedures. The files of each system were used to prepare only one canal, and a total of 15 mL of 2.5% sodium hypochlorite solution (NaOCl) was used per canal.

### Twisted Files Adaptive group

The medium/large sequence (ML1: #25/.08; ML2: #35/.06; ML3: #50/.04) was employed with gentle pressure and brushing motion within the "TF Adaptive program" on the Elements motor (SybronEndo, Orange, CA, USA), following the manufacturer's instructions. After reaching the working length (WL), each instrument was subsequently withdrawn, cleansed, and followed by irrigation, aspiration, and inundation of the canal with 5 mL of 2.5% NaOCl solution, using a conventional syringe and 30G needle positioned apically without contacting the root walls (Navitip, Ultradent, South Jordan, UT, USA). The patency of the canal was maintained throughout the experimental procedure using a #10 K-file (Dentsply Maillefer, Ballaigues, Switzerland).

### Reciproc group

According to the manufacturer's instructions, the Reciproc R50 (50/.05) instrument was used in a reciprocating motion by a VDW Silver electric motor (VDW GmbH, Munich, Germany). The file was gradually inserted in the canal using an in-and-out pecking motion of approximately 3 mm amplitude with lateral brushing strokes. After three pecking motions, the instrument was removed and cleaned, the canal was irrigated, aspirated, and flooded with 5 mL of 2.5% NaOCl, using a conventional syringe and 30G needle positioned apically without contacting the root walls (Navitip, Ultradent, South Jordan, UT, USA. A #10 K-file was used to ensure patency. This movement was repeated until the file reached the WL.

After preparation, in the TFA and RC groups, the canals were aspirated with a Capillary Tip suction cannula (Ultradent, South Jordan, UT, USA) and dried with sterile absorbent paper points 50/.04 or 50/.05, respectively. For the second scanning (post-preparation), the pulp chamber was sealed with a temporary sealer, and the polyvinyl siloxane was removed.

### Final Irrigation

In both groups, a final UI was performed. The solution was activated using an ultrasonic tip with a 0,20mm tip and a 0,01mm taper (Irrisonic, Helse Dental Technology, São Paulo, Brazil), positioned 2 mm from the working length, and allowed to vibrate freely at minimum power. The following protocol was applied: irrigation with 2 ml 2.5% NaOCl followed by activation for 20 seconds (3x); irrigation with 2 ml 17% EDTA followed by activation for 20 seconds (3x); and once again irrigation with 2 ml 2.5% NaOCl followed by activation for 20 seconds (3x). Subsequently, the root canals were aspirated with a Capillary Tip suction cannula, dried with a sterile absorbent paper point, depending on the sample group, and the pulp chamber was sealed with a temporary sealer. Finally, the third scanning (post-UI) was performed.

### Micro-CT Scanning and Analysis

The samples were scanned three times for analysis: pre-preparation, post-preparation, and post-UI. A micro-CT device (SkyScan 1176; Bruker-microCT, Kontich, Belgium) was used, and the parameters were set at 70 kV, 279 µA, 360° rotation, 0.3° rotation step, and an isotropic resolution of 8,71 µm. NRecon v. 1.7.1.6 software was used to reconstruct the images, and DataViewer v. 1.5.6 software (Bruker-microCT, Kontich, Belgium) was used to register and geometrically align the three image sets. CTan v.1.12.0.0 software allowed a quantitative analysis of: a) the change in volume by subtracting the post-preparation root canal volume from the pre-preparation volume ^14^ ^,^ ^21^; b) the amount of unprepared root canal surface by calculating the number of static voxels, expressed as a percentage of the total number of voxels on the canal surface ^1^ ^,^ ^17^; c) the AHTD after root canal preparation ^9^ ^,^ ^14^ ^,^ ^20^ by the presence of material with a density similar to dentin in the images post-preparation in areas that were previously occupied by air in the canal before preparation and d) the AHTD reduction after-UI identified by the images intersection after-UI and hard-tissue debris post-preparation ^20^.

CTVol v.2.2.1.0 software (Bruker-microCT, Kontich, Belgium) allowed manipulating 3D surface rendered models created in the CTan software, making tangible aspects of the 3D structure of the objects, which permitted the comparison of superimposed root canal models from the three scans by a blinded examiner. A color-coded standard for the root canals was used - preoperative root canals in green and postoperative in red. The AHTD was colored black over the postoperative anatomy ^20^.

All calculations were performed for the entire canal and each third: cervical, middle, and apical.

### SEM analysis

After the final micro-CT scan, five samples from each group that presented the apical third with the most significant percentage reduction in AHTD after UI were evaluated using SEM images. The samples were split into two halves longitudinally, following the foramen opening. The specimens were fixed in 2% glutaraldehyde and dehydrated in a graded series of ethanol solutions. Further on, they were dried, mounted on stubs, gold-coated, and examined by SEM (LEO 430i; LEO Electron Microscope, Cambridge, England). All images were taken at 1000x magnification, and the smear layer distribution was qualitatively evaluated by blinded examiners (LGF; SKB; Kappa test = 0.61) with the scoring system proposed by Hülsmann et al. (1997) ^22^, which evaluates the presence and distribution of the smear layer as follows: score 1 - absence of smear layer, with open dentinal tubules; score 2 - a small amount of smear layer, with some open dentinal tubules; score 3 - represents a homogeneous smear layer covering the canal wall, with only a few open dentinal tubules; score 4 indicates the root canal wall completely covered by a homogeneous smear layer, with no open dentinal tubules; and score 5 denotes a thick, nonhomogeneous smear layer completely covering the root canal wall.

### Statistical analysis

Statistical analysis revealed that the data samples exhibited a non-normal distribution as determined by the D’Agostino test using the BioEstat software v.5.3 (Institute of Sustainable Development Mamiraua, Amazonas, Brazil). The Mann-Whitney test was used for inter-group analysis, while the Kruskal-Wallis test was employed for intra-group comparisons. The significance level was set at p<0.05. For SEM image analysis, the presence of the smear layer was assessed using a categorical score system. To evaluate inter-examiner agreement for these scores, Cohen's Kappa test was applied (k=0.61).

The correlation between the percentage reduction of hard tissue debris obtained by micro-CT and the smear layer scores from SEM analysis was determined using Pearson's correlation coefficient. The data were normalized and tested for homoscedasticity using a computer programming platform. The statistical analysis was performed using the MATLAB programming platform (MathWorks, Boston, USA).

## Results

The initial volume of the distal root canal showed no significant differences between groups TFA and RC (p>0.05). In both groups, the action of each system increased volume in the whole and each third of the root canals (Table 1). The final volume of the entire canal was superior in TFA than in the RC group (*p<0.05*).

**Table 1:** Mean and standard deviation of morphometric alterations in volume (mm^3^), surface area (mm^2^), and percentage of unprepared surfaces. Mean and standard deviation of AHTD volume (mm^3^) after root canal preparation, after-UI, and the percentage of reduction after-UI for TFA and RC groups.

	Region	TFA	RC
initial canal volume (mm^3^)	Entire	7.296 ± 1.040	6.859 ± 0.958
	Cervical	3.933 ± 0.866	4.143 ± 0.716
	Medium	2.069 ± 0.614	1.963 ± 0.420
	Apical	1.255 ± 0.665	0.954 ± 0.510
final canal volume (mm^3^)	Entire	13.136 ± 1.796^A^	11.086 ± 1.463^B^
	Cervical	7.393 ± 1.547	6.605 ± 1.254
	Medium	3.459 ± 1.054	3.071 ± 0.684
	Apical	2.053 ± 1.441	1.644 ± 0.871
initial surface area (mm^2^)	Entire	55.131 ± 13.348	46.688 ± 10.907
	Cervical	27.110 ± 8.057	23.872 ± 6.333
	Medium	20.956 ± 4.659	18.414 ± 10.256
	Apical	7.098 ± 3.296	7.717 ± 4.971
final surface area (mm^2^)	Entire	68.080 ± 17.338	63.824 ± 15.808
	Cervical	33.959 ± 10.622	30.947 ± 9.114
	Medium	23.276 ± 6.543	24.451 ± 13.291
	Apical	10.394 ± 3.700	8.362 ± 3.377
unprepared Surface (%)	Entire	26,767 ± 13,497	29,214 ± 10,096
	Cervical	19,058 ± 12,068	21,763 ± 11,878
	Medium	33,738 ± 17,799	33,922 ± 15,619
	Apical	37,083 ± 21,513	44,852 ± 17,241
AHTD after preparation	Entire	0.274 ± 0.302^a^	0.349 ± 0.343^a^
	Cervical	0.078 ± 0,113^c^	0.139 ± 0.263^c^
	Medium	0.088 ± 0.085^e^	0.119 ± 0.132^e^
	Apical	0.085 ± 0.106^g^	0.084 ± 0.065^g^
AHTD after-UI	Entire	0.127 ± 0.152^b^	0.134 ± 0.130^b^
	Cervical	0.022 ± 0.041^d^	0.042 ± 0.049^d^
	Medium	0.043 ± 0.058^f^	0.050 ± 0.063^f^
	Apical	0.037 ± 0.051^g^	0.034 ± 0.035^g^
AHTD Reduction after-UI (%)	Entire	71.778 ± 17.146	63.543 ± 25.384
	Cervical	71.117 ± 18.936	65.582 ± 27.842
	Medium	69.376 ± 25.495	63.759 ± 27.256
	Apical	67.487 ± 16.570	63.440 ± 24.209

No significant differences between experimental groups (p>0,05)

Different uppercase letters show significant differences between groups (p<0,05)

Different lowercase letters show intra-group significant differences (p<0,05)

An increase in surface area was observed for the whole of the root canal and each third in both groups, but without a significant statistical difference (Table 1) (*p>0.05*).

After preparation, both systems left unprepared canal walls with no statistical difference between groups. TFA and RC instruments showed approximately 26 to 29% of the surface area unprepared for the whole canal (Table 1).

**Figure 1:**
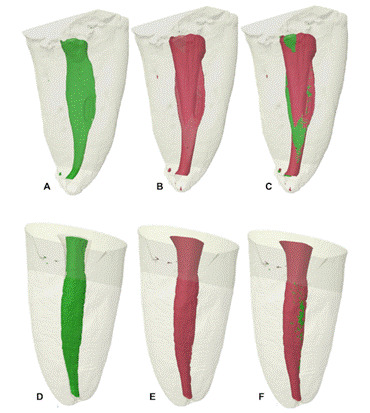
Representative 3D reconstructions of the mandibular distal canal of teeth prepared using TFA (A-C) and Reciproc (D-F) systems. In A and D: before root canal preparation; In B and E: after root canal preparation; In C and F: overlay of A or D (green), and B or E (red) images.

**Figure 2:**
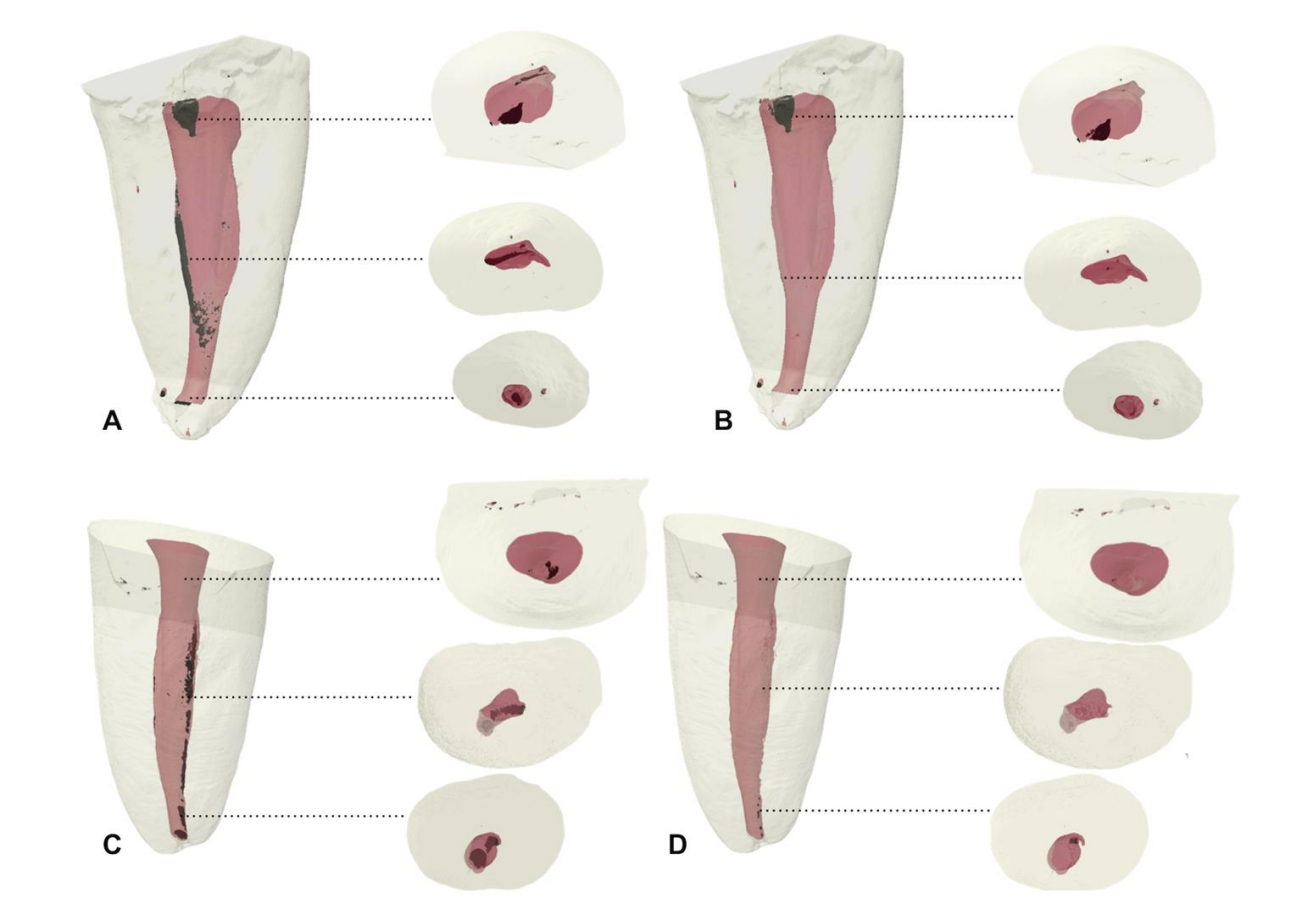
Representative 3D reconstructions of the mandibular distal canal of teeth prepared using TFA (A and B) and Reciproc (C and D) systems after UI. In A and C: AHTD (black) after preparation; In B and D: AHTD post-UI. Dotted lines correspond to cross-sectional views of each third of the root canal.

Regarding the AHTD, Table 1 shows the mean volume of AHTD after preparation and after UI, and the percentage of AHTD reduction after UI. The TFA and RC groups presented AHTD volume after root canal preparation from 0.274 to 0.349 mm³ (*p>0.05*), and a statistically significant reduction after UI (71% to 63%, respectively) was observed for both systems (Table 1). The reduction after UI was substantial in all thirds except for the apical third. Figures 1 and 2 display representative 3D reconstructions of the distal canal of each experimental group, which showed the highest percentage of hard-tissue debris reduction after UI in the cervical third.

**Figure 3:**
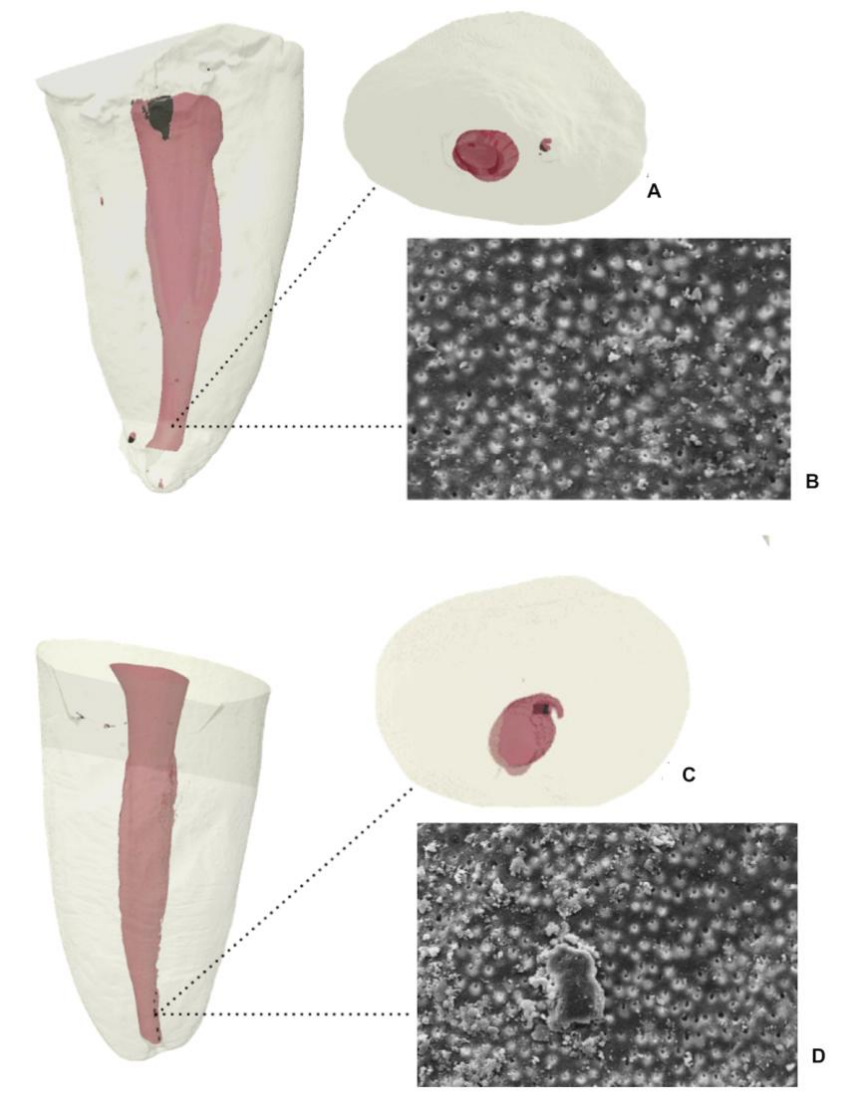
3D models of the sample of each group (A and B - TFA; C and D - RC) with the highest AHTD reduction after ultrasonic irrigation in the apical third seen throughout micro-CT (A and C) and the representative microphotograph from SEM B and D. SEM images were obtained using the secondary electron (SE) detector at a magnification of x1000, and a scale bar of 10 micrometers.

The SEM analyses demonstrated the smear layer accumulated in the apical third of samples from each experimental group. Pearson's analysis revealed a strongly significant negative correlation between the quantification of hard tissue debris by micro-CT and the smear layer scores obtained by SEM analysis (r=-0.91). In the TFA group, the specimen demonstrated a reduction of AHTD from 51 to 84% and moderate to heavy smear layer - scores 2 and 3 - and the Reciproc group presented a reduction of AHTD from 39 to 88% and heavy to complete smear layer - scores 3 and 5 (Table 2). Pearson’s correlation revealed that the higher percentages of hard tissue debris removal observed with micro-CT were associated with lower smear layer score in SEM, suggesting better dentinal surface cleanliness. Figure 3 shows representative microphotographs of both systems.

**Table 2:** Mean and standard deviation of the accumulated hard tissue debris (AHTD) in the apical third for TFA and RC groups, and the reduction (%) after UI obtained by micro-CT, and smear layer median scores obtained by SEM.

	TFA	RC
AHTD after preparation	0.085 ± 0.106	0.084 ± 0.065
AHTD after-UI	0.037 ± 0.051	0.034 ± 0.035
AHTD Reduction after-UI (%)	67.487 ± 16.570	63.440 ± 24.209
Predominant SEM scores	2.5 (2-3)	4 (3-5)

r=- 0.91. p<0.01

## Discussion

The null hypothesis of the present study was rejected, as the TFA system demonstrated superior efficiency in dentin removal compared to the RC system, and the final UI promoted an additional reduction in AHTD. These findings highlight the influence of the instrumentation system and supplementary activation techniques on the preparation and cleanliness of long-oval root canals.

Although both systems presented similar percentages of unprepared root canal surfaces, the root canal volume increase was more significant for the TFA system than the RC system (p< .05), suggesting that the TFA removed more dentin than the RC files, even though the TFA presented the smallest taper. This difference may be attributed to the fact that the TFA system uses multiple files, whereas the RC system is a single-file system, potentially affecting the efficiency of dentin removal ^3^ ^,^ ^23^. These results confirm that an increased canal volume does not indicate a more prepared root canal surface ^6^ ^,^ ^20^ ^,^ ^21^ ^,^ ^22^ ^,^ ^23^ ^,^ ^24^ and suggest that the Twisted File technology can improve the root canal preparation.

On the other hand, the AHTD was not statistically different between groups. As a collateral effect of the instrument's action on the canal wall, hard-tissue debris production should come under the spotlight of the TFA system that increases the canal volume, probably creating more AHTD. However, in contrast, TFA instruments left the same amount of AHTD as the RC system, despite some studies showing that the reciprocating cinematic presented more AHTD than rotary movement ^9^ ^,^ ^15^ ^,^ ^25^. A previous study also found no difference in AHTD between the TFA system and the rotary system Protaper Next (Dentsply Maillefer, Ballaigues, Switzerland) ^26^. The results of this investigation supported the conclusion that system kinematics did not influence the amount of AHTD in the root canal, just as in previous studies ^6^ ^,^ ^16^ ^,^ ^24^ ^,^ ^27^ ^,^ ^28^.

Dentinal debris can harbor microorganisms and prevent the irrigant flow, neutralizing its bacterial effect ^18^. Clinical repercussions of AHTD are unclear ^10^, but methods for irrigant solution activation have been the focus of several investigations to improve cleaning and disinfection of the root canal system. In vitro studies have shown that ultrasonic irrigation is more efficient than conventional irrigation in AHTD removal ^18^ ^,^ ^27^ ^,^ ^29^ ^,^ ^30^. Ultrasonic irrigation and micro-CT evaluation have been used in previous studies ^9^ ^,^ ^21^ ^,^ ^26^ ^,^ ^29^ ^,^ ^30^ due to the importance of this methodology in analyzing hard tissues in three dimensions. Based on micro-CT observation, UI reduced 63 to 71% of AHTD in the present study. In previous reports in canal models with an isthmus using UI, the reduction was around 50% because of the irrigant's difficulty in reaching isthmus areas ^29^ ^,^ ^31^. However, micro-CT cannot evaluate what is adhered to the root canal wall, and no study has correlated the AHTD identified by micro-CT with another method of analysis that determines the smear layer. For this reason, SEM was used to relate the presence of the smear layer to areas of the most excellent AHTD removal observed by micro-CT.

Many papers evaluated canal preparation and the presence of AHTD using micro-CT, while others assessed the presence of smear layer by SEM ^28^ ^,^ ^32^ or vital pulp tissue by histologic analysis ^17^ ^,^ ^18^. Nevertheless, to the best of our knowledge, this study related the cleaning of long-oval canals using two experimental methods: micro-CT for AHTD and SEM for the smear layer. Micro-CT analysis is a nondestructive method that allows the examination of the entire root canal anatomy and the different stages of root canal treatment, but it only provides information about hard tissues. SEM analysis offers a much higher resolution and direct visualization of the smear layer and the dentinal tubule with fine surface details of the smear layer, including both hard and organic tissues, though it is a destructive technique. Combining both methods can provide a more complete understanding of the effects of irrigation on cleaning the root canal walls.

The TFA group demonstrated, in areas of 51 to 84% AHTD reduction, a comparatively small amount of homogeneous smear layer covering the canal wall (scores 2 and 3). While the RC group showed homogeneous to heavy nonhomogeneous smear layer covering the complete canal wall (scores 3 and 5), and areas of 39 to 88% AHTD reduction. Pearson's correlation revealed that the higher percentages of hard tissue debris removal observed with micro-CT were associated with lower smear layer score in SEM, suggesting better dentinal surface cleanliness. Whereas micro-CT analysis showed a substantial reduction of AHTD, SEM evaluation revealed the persistent presence of the smear layer. The study also sought to verify whether the correlation between micro-CT data and SEM smear layer scores existed, validating whether these two approaches converge in their assessment of the root canal cleanliness. The establishment of such a relationship would be relevant since micro-CT, if proven consistent with SEM findings, could present a valuable methodological alternative to conventional tooth cleavage. Avoiding potential artifacts and interferences that may arise from specimen sectioning or splitting for the evaluation of dentinal debris removal and smear layer presence. Although a strong negative correlation was observed between the quantification of hard tissue debris by micro-CT and the smear layer scores obtained by SEM analysis, the assessment of dentinal tubule openness can only be performed by SEM analysis. This discrepancy is important to highlight that when micro-CT reveals a root canal relatively clean in terms of hard tissue debris, images from SEM exhibit dentinal tubules wholly or partially occluded by smear layer, a key factor for achieving root canal cleanliness and ensuring the success of the endodontic therapy.

Micro-CT scanning was performed in high resolution (8.71µm) for more sensitive detection of unprepared surfaces and the presence of AHTD. The resolution is related to a) the minimum size of the voxel necessary to be removed so the surface is considered touched/prepared ^24^ and b) discerning the presence of material with a density like dentin in post-preparation images in areas previously occupied by air in the pre-preparation root canal - assigned as hard-tissue debris ^10^ ^,^ ^33^. The applied resolution was chosen to determine precise outcomes. A previous study suggests a tendency to have a higher percentage of debris in analyses performed at a smaller voxel size than those performed at a larger voxel size ^30^. The majority of the studies related to untouched canal walls and AHTD removal employ a resolution lower than 8.71µm ^6^ ^,^ ^9^ ^,^ ^20^ ^,^ ^26^ ^,^ ^27^, probably because of the scanning and analysis time under higher resolution, which can be two to three times higher compared to lower resolutions.

New root canal preparation techniques and changes in the kinematics of mechanized NiTi instruments are continually emerging, potentially offering improvements over previous methods. Nevertheless, the initial morphology of the canal system has a more significant impact on the quality of preparation than the instrumentation techniques themselves ^21^ ^,^ ^31^. Long oval canals' buccal and lingual recesses are particularly challenging for instruments to access. These unprepared recesses can harbor dentin debris, smear layer, and untouched root canal walls ^6^ ^,^ ^7^, compromising disinfection and adversely affecting endodontic treatment outcomes. For this reason, we investigated the performance of two endodontic systems, Twisted File Adaptive and Reciproc, in preparing long-oval distal canals of mandibular molars, measuring the amount of AHTD after the root canal preparation and its reduction after ultrasonic irrigation.

Exploiting knowledge on the clinical implications of shaping and cleaning long-oval canals is still a challenge in root canal therapy. Both systems accumulated hard-tissue debris similarly and were not able to touch all surfaces of an oval-shaped canal completely. Ultrasonic irrigation significantly reduces AHTD, highlighting its potential in canal cleaning.

One limitation of this study is that the SEM analysis was restricted to the apical 5 mm of the root canal. Therefore, the findings may not fully represent the entire root canal system, and caution is needed when extrapolating the results.

## Conclusion

Within the limitations of this ex vivo study, it can be concluded that the use of reciprocating and adaptive motion systems did not significantly affect the amount of unprepared surfaces or accumulated hard-tissue debris (AHTD) following the preparation of long-oval distal root canals in mandibular molars. However, the final ultrasonic irrigation was effective in reducing the amount of AHTD. Additionally, a strong, significant negative correlation was observed between the presence of AHTD, as assessed by micro-CT, and the smear layer detected through SEM.

## Data Availability

The research data are available upon request.
